# Effect of middle turbinate-septal suturing technique on success of endoscopic dacryocystorhinostomy

**DOI:** 10.1097/MD.0000000000042057

**Published:** 2025-06-20

**Authors:** Kamil Gokce Tulaci, Tugba Tulaci

**Affiliations:** a Departments of Otorhinolaryngology Head and Neck Surgery, Faculty of Medicine, Balikesir University, Balikesir, Turkey.

**Keywords:** endonasal dacryocystorhinostomy, granulation, nasolacrimal duct obstruction, silicon stent, synechia

## Abstract

This study investigates the effect of the middle turbinate-septal suturing technique on postoperative synechiae formation and surgical success in endoscopic endonasal dacryocystorhinostomy. A total of 98 patients aged between 18 and 65 years who underwent endoscopic endonasal dacryocystorhinostomy and silicone tube intubation for nasolacrimal duct obstruction were included. The nonsuture group consisted of patients in whom the middle turbinate-septal suture procedure was not performed, and the suture group consisted of patients in whom the middle turbinate-septal suture procedure was performed. The rates of postoperative synechiae, granulation tissue formation, anatomical success, and functional success were compared between groups. The nonsuture group consisted of 45 patients and the suture group consisted of 53 patients. The comparison of postoperative synechiae and granulation tissue formation revealed that both synechiae and granulation tissue formation were significantly lower in suture group compared with nonsuture group (*P* = .046 and.04, respectively). In addition, both anatomical and functional success rates were significantly higher in the suture group than in the nonsuture group (*P* = .019 and .025, respectively). The results suggest that suturing the middle turbinate to the nasal septum at the conclusion of surgery diminishes the incidence of postoperative synechiae and positively affects surgical outcomes.

## 1. Introduction

Dacryocystorhinostomy (DCR) is a surgical procedure commonly used to treat nasolacrimal duct (NLD) obstruction. The main goal of DCR is to create a new passage between the nasal cavity and lacrimal sac located proximal to the NLD. DCR can be performed using both the endoscopic and external approaches. The endoscopic approach offers several advantages, including the absence of a visible scar, reduced surgical time, more comfortable and expedited recovery, preservation of the medial canthal tendon, and the ability to address other nasal pathologies simultaneously.^[1]^ Surgical failure in endoscopic endonasal DCR (EDCR) ranges from 4% to 24%.^[2]^

EDCR surgery failure can be attributed to numerous factors, including intraoperative complications, such as bleeding, misplaced fistula openings, inadequate mucosal or bony patency, and insufficient space for normal function. Postoperative complications, such as crusting, synechiae, granulation tissue formation, and stenosis, can also contribute to failure.^[1,3]^

According to previous reports, the most common reasons for failure in EDCR and the need for revision surgery were postoperative granulation tissue formation and synechiae formation between the neo-ostium site and the middle turbinate.^[1,4,5]^

To date, various methods have been used to prevent synechiae formation, including the use of antiadhesion adjuvants, biodegradable materials, substances such as mitomycin C, trimming the anterior portion of the middle turbinate, and intraoperatively created nasal mucosal flaps.^[1,6–9]^ Despite the implementation of diverse surgical techniques and adjunctive materials, ongoing research aims to determine an optimal approach for minimizing the risk of EDCR failure by preventing the formation of synechiae and granulation tissue.^[1]^

Functional endoscopic sinus surgery (FESS) is a minimally invasive surgical technique that shares many similarities with EDCR in terms of the surgical instruments employed, surgical field, and underlying surgical principles. The FESS serves as the basis for various endoscopic nasal surgeries. Prior studies demonstrated that suturing the middle turbinate to the nasal septum (middle turbinate-septal suturing technique) at the conclusion of the FESS can effectively reduce postoperative synechiae formation and increase surgical success.^[10–12]^

To the best of our knowledge, previous studies have not investigated the use of the middle turbinate-septal suturing technique in EDCR. Therefore, the goal of this study was to assess the effects of this technique on postoperative synechiae formation and overall success of the EDCR procedure.

## 2. Materials and methods

### 2.1. Subjects and study design

This study was conducted in accordance with the Declaration of Helsinki. Approval was obtained from the approval from the Balikesir University Ethics Committee (approval number: 2024/159). The demographic and clinical data of the patients were collected from their medical records. All patients were provided with comprehensive information about the study, agreed to participate in the research, and provided written informed consent for review of their medical records.

Patients aged 18 to 65 patients who underwent EDCR and silicone tube intubation for NLD obstruction between July 2018 and December 2023 were included in this retrospective study.

The exclusion criteria included pediatric patients; patients with bilateral NLD obstruction; punctum stenosis with NLD obstruction; ectropion or lacrimal hypersecretion; a previous history of DCR; systemic diseases including diabetes mellitus or hypertension; concurrent or previous turbinate surgery, septoplasty, or FESS; rhinitis; a history of nasal, paranasal, or ocular trauma or tumor; incomplete medical records; and those who did not attend regular follow-up visits.

All patients who underwent EDCR were referred from the ophthalmology clinic because of epiphora secondary to NLD obstruction. The decision to perform surgery was based on the identification of NLD obstruction through physical examination findings, lacrimal syringing, and confirmation of NLD obstruction with dacryoscintigraphy. All patients underwent a complete otorhinolaryngological and ophthalmological examination before surgery. Preoperatively, paranasal sinus computed tomography was performed in all patients.

The patients were divided into 2 groups based on the implementation of the middle turbinate-septal suture procedure, with July 2021 serving as the cutoff point. Before this date, the suturing technique was not used in the clinic where the study was conducted; thus, patients who underwent surgery before July 2021 were classified into the nonsuture group (patients in whom the middle turbinate-septal suture procedure was not performed). Following July 2021, the suturing technique became routine practice, and these patients were categorized into the suture group (patients in whom the middle turbinate-septal suture procedure was performed). These groups were compared in terms of postoperative synechiae formation and the overall success of the EDCR procedure.

### 2.2. Surgical method

All surgeries were performed by the same surgical staff using the same surgical method. It was performed under general anesthesia using a 0° 4-mm nasal endoscope (STORZ) in the standard endoscopic sinus surgery position. The surgical procedure was performed after local infiltration with lidocaine hydrochloride + epinephrine (Jetocaine^R^ 20 + 0.0125 mg/mL) applied to the middle turbinate attachment site, anterior section of the uncinate process, lacrimal sac projection, and sphenopalatine artery localization at the junction of the middle turbinate with the lateral nasal wall. The surgical procedure was initiated 10 minutes after infiltration. A mucosal incision was made starting approximately 6 to 8 mm above the attachment site of the middle turbinate and continuing downwards, followed by posterior flap elevation. Then, using Kerrison forceps, a bone window of approximately 1 × 1 cm was opened from the posterior part of the frontal process of the maxilla and the lacrimal bone. The lacrimal sac was identified after the bone window was opened. A punctum dilator was used to dilate the puncta. Subsequently, lacrimal probing was performed, and the medial wall of the lacrimal sac was visible with a tenting effect. An incision was made in the sac from top to bottom, and the lateral mucosa of the sac was made visible by creating anterior and posterior flaps on the medial wall of the sac. The flaps created from the medial mucosa of the sac were excised using a microdebrider to create a wide opening. A lacrimal syringe test was performed, fluid passage was observed, and anatomical patency was confirmed.

**Figure 1.**
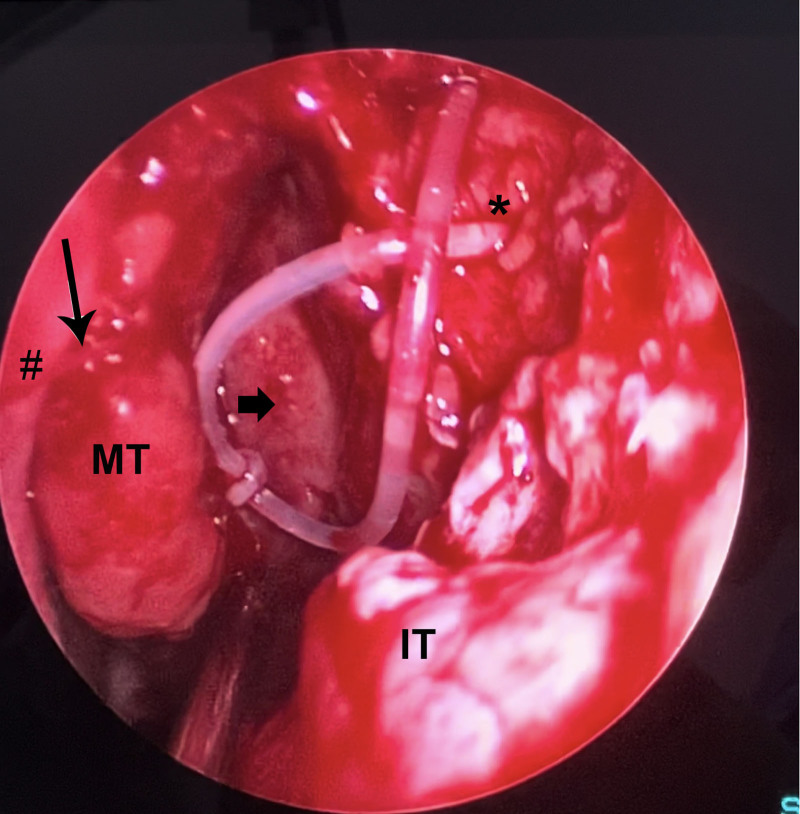
The left nasal cavity, demonstrating that suturing the middle turbinate to the septum prevents direct contact between the bare surfaces of the neo-ostium and the middle turbinate. IT = inferior turbinate, MT = middle turbinate, thick arrow = ethmoid bulla, thin arrow = suture of the middle turbinate, *nasolacrimal sac, **#**nasal septum.

The DCR stent (Teknomek^R^ DCR Set: Stainless steel probes: 4.5 cm length, with a 45° bend 15 mm from the tip. Solid silicone tubing (40 cm in length) was applied. Surgery was terminated in patients in whom suturing of the middle turbinate was not performed after stenting. In patients in whom the middle turbinate was sutured, suturing was performed using a 4-0 vicryl rapid (ETHICON^R^) suture with a 19-mm needle, following the stent procedure. For the suturing process, the needle first started from the lateral middle turbinate, passed through the middle turbinate, passed through the nasal septum, and reached the opposite nasal passage. The needle was passed through the nasal septum again, passed to the nasal cavity where surgery was performed, and sutured under endoscopic vision in front of the middle turbinate. The operation was terminated without nasal packaging (Fig. 1).

### 2.3. Postoperative care and follow-up

Nasal irrigation with nasal saline solution (by using plastic bottles designed for nasal irrigation [squeeze bottle, 240 mL]) was initiated in the patients 6 hours after the surgery for the care of the surgical area, and it was recommended to do it 4 times a day for 4 weeks. On the morning of the first day after the operation, lacrimal syringing was performed by an ophthalmologist, patency was reevaluated, and the patient was externalized. Systemic antibiotics (2 times per day for 1 week), systemic anti-inflammatory and analgesic agents (2 times per day), antihistamine tablets (once per day), and intranasal corticosteroids were prescribed. The patients had routine outpatient clinic controls at the 1st week, 1st month, 3rd month, and 6th month. All stents were removed in the 3rd month.

Surgical success was evaluated in 2 separate ways: anatomical and functional success.

#### 2.3.1. Anatomical success

Under endoscopic nasal examination, when the lacrimal sac projection was palpated externally, an opening between the lacrimal sac and the nasal cavity was observed along with the irrigation fluid in the nasal cavity during the lacrimal irrigation (syringe) procedure.

#### 2.3.2. Functional success

Functional success was evaluated using the Munk scale. For functional success measurement on the Munk scale, a score of 1 or less is considered functional success, and a score of 2 or more is considered functional failure^[13,14]^ (Table 1).

**Table 1:** Munk scale.

Grade	Munk scale
0	No sign of epiphora
1	Epiphora that requires eye-rubbing less than 2 times a day
2	Epiphora that requires 2–4 times of eye-rubbing
3	Epiphora that requires 5–10 times of eye-rubbing
4	Epiphora that requires eye-rubbing at least 10 times a day
5	Constant epiphora

Nasal synechiae (between the middle turbinate and lateral nasal wall- neo-ostium) and granulation tissue were monitored via an endoscopic nasal examination.

### 2.4. Sample size calculation

The sample size was calculated using the G power 3.1.9 program to evaluate whether the functional and anatomical success of endoscopic DCR would create a statistically significant difference between the 2 groups with and without suturing the middle turbinate to the nasal septum by chi-square (χ²) test. In the calculation, it was concluded that a minimum of 88 participants was required at medium effect size (effect sizes for the chi-square (χ²) test according to Cohen 0.1 = small effect size, 0.3 = medium effect size, 0.5 = large effect size) when type I error (α) = 0.05 and type II error (1-β) = 0.80 were accepted.^[15]^ Therefore, the study was planned to be conducted with 98 patients.

### 2.5. Statistical analysis

The data of the present study were evaluated using the Statistical Package for Social Sciences for Windows (version 22.0; SPSS Inc., Chicago, IL) software. Descriptive statistics are expressed as mean (±) standard deviation, frequency distribution, and percentage. The distribution normality of variables was evaluated using the Kolmogorov–Smirnov test. Categorical variables were evaluated using Chi-square and Fisher’s exact tests. The Mann–Whitney *U* test was used for the data analysis of continuous variables. Statistical significance was set at *P* <.05.

## 3. Results

A total of 118 patients aged between 18 and 65 years who met the inclusion criteria were initially evaluated for the study. However, 12 patients in the nonsuture group and 8 patients in the suture group were excluded because of irregular follow-up visits. Ultimately, 98 patients with complete medical records were included in the final analysis. Among the patients, 26 (26.5%) were male and 72 (73.5%) were female. The mean age of the patients was 56.5 ± 15.3 years. The mean follow-up period of the 98 patients included in the study was 17.1 ± 7.1 months.

The nonsuture group consisted of 45 patients and the suture group consisted of 53 patients. No significant differences were found among the groups with respect to age, sex, and follow-up period (*P* = .471, *P* = .373 and *P* = .165, respectively) (Table 2).

**Table 2:** Demographics of patients, postoperative complications, success rates, and other clinical information.

	Nonsuture group (n = 45)	Suture group (n = 53)	*P*-value
Age*	58.0 ± 15.6	55.2 ± 15.2	.471†
Gender (F/M)	35/10	37/16	.373‡
Synechiae (±) (%)	6/39 (13.3%)	1/52 (1.9%)	.046‡
Granuloma (±) (%)	8/37 (17.8%)	2/51 (3.8%)	.040‡
Anatomically successful, n (%)	35 (77.8%)	50 (94.3%)	.019‡
Functional successful, n (%)	33 (73.3%)	48 (90.6%)	.025‡
Deviation, n (%)	8 (17.8%)	14 (26.4%)	.307‡
Side (right/left)	23/22	31/22	.460‡
Revision	4 (8.9%)	2 (3.8%)	.409‡
Follow-up (month)	18.2 ± 7.6	16.0 ± 6.5	.165†

F = female, M = male, n = patient number.

* Presented as mean ± standard deviations, + denotes present, − denotes absent.

† Mann–Whitney *U* test.

‡ Chi-square test.

Comparison of postoperative synechiae and granulation tissue formation revealed that the rates of synechiae and granulation tissue formation were significantly lower in the suture group than in the nonsuture group (*P* = .046 and .040, respectively) (Table 2).

Moreover, both the anatomical and functional success rates were significantly higher in the suture group than in the nonsuture group. (*P* = .019 and .025, respectively) (Table 2). A detailed comparison of the patients who experienced anatomical and functional failure is presented in Table 3.

**Table 3:** Characteristics and failure reasons of patients with anatomical or functional failure.

Failure type	Nonsuture group (n = 45)	Suture group (n = 53)	Main failure reasons
Anatomical failure	10 (22.2%)	3 (5.7%)	Granuloma (7) Synechiae (4) Stent dislocation (2)
Functional failure	12 (26.7%)	5 (9.4%)	Granuloma (9) Synechiae (6) Stent dislocation (2)

n = patient number.

Revision surgery was performed in 4 (8.9%) patients in the nonsuture group and in 2 (3.8%) patients in the suture group.

An evaluation of the reasons for revision surgery revealed that in the nonsuture group, 2 patients required revision due to granulation tissue formation, and 2 patients underwent revision for complete synechiae between the middle turbinate and the lateral nasal wall. In the suture group, 1 patient required revision due to granulation tissue formation and another underwent revision because the silicone stent had become fully embedded in the mucosa of the lateral nasal wall, resulting in dislocation.

## 4. Discussion

This study investigated the effects of the middle turbinate – septal suturing technique at the conclusion of EDCR surgery to prevent postoperative synechiae formation (one of the most common causes of EDCR failure) and on the success of EDCR surgery. According to our literature review, this is the first study investigating the use of the middle turbinate-septal suturing technique, which has been shown to be effective in preventing postoperative synechiae formation and increasing surgical success in FESS, to prevent postoperative synechiae formation in EDCR.^[10–12,16]^ Our results showed that suturing the middle turbinate to the nasal septum at the conclusion of the surgery reduced postoperative synechiae.

EDCR has become a widely preferred surgical method for the treatment of NLD obstruction with recent developments in endoscopic systems and endoscopic sinus surgery. The final goal of a successful EDCR is to create a new fistula between the nasolacrimal sac and nasal cavity to provide permanent drainage. EDCR has some advantages against external technique such as being a minimally invasive procedure, shorter operative time, no skin scarring, and faster healing time. Moreover, the long-term functional and anatomical success rates of EDCR have been reported to range from 72% to 96%, similar to external DCR.^[2]^

The most common factors causing failure in EDCR are obstruction of the newly opened ostium due to complications such as granulation tissue and synechiae formation between the neo-ostium and middle turbinate.^[1,17–20]^ The frequency of synechiae and granulation tissue formation after surgery varies between 0.6% and 11%.^[21]^

Various methods have been employed to prevent postoperative synechiae and ensure long-term function of the fistula. These methods include placing a pack between the middle turbinate and the lateral nasal wall, partial resection of the middle turbinate, stent placement, and application of substances such as antiadhesive and mitomycin C. However, postoperative synechiae still occurs even in surgeries performed by the most experienced surgeons, affecting surgical success.

A review of studies exploring supplementary techniques to enhance EDCR outcomes and minimize postoperative complications revealed that Prakash et al employed an approach by trimming the anterior tip of the middle turbinate to prevent synechiae and granulation tissue formation. Their strategy yielded a remarkable success rate of 94.6%, consistent with the existing literature, and mirroring the findings of our study. In their series of 37 patients, only 2 failures occurred. One was canaliculitis, and the other was granulation tissue formation. Prakash et al reported that the reason for the absence of synechiae in their study was the prevention of contact between the middle turbinate and the lateral nasal wall.^[17]^ However, although partial middle turbinate resection in FESS was previously shown to reduce synechiae formation, this technique has been abandoned over the years because of the functional importance of the middle turbinate in humidifying inhaled air and creating laminar airflow.^[10,11]^

Therefore, based on our results, the middle turbinate-nasal septum suturing technique may be considered a more conservative and feasible method than partial resection of the middle turbinate.

A study of Ceylan SM focusing on the use of fibrin glue to prevent synechiae and granulation tissue formation in the surgical field revealed that fibrin glue minimized the complication risk and increased the surgical success compared with cases in which fibrin glue was not used.^[22]^ However, fibrin glue has a cost burden of approximately 140 USD per case. Therefore, the middle turbinate-septal suturing method offers a more economical option as it is an important advantage against the use of supplementary materials such as fibrin glue. Moreover, the suturing technique retains its advantage even in cases requiring septoplasty along with EDCR because the suture materials used in septoplasty can also be used between the middle turbinate and septum without any additional cost.

Although previous studies have demonstrated the potential benefits of mitomycin C application, conflicting opinions exist regarding its efficacy in achieving positive anatomical and functional outcomes. Consequently, the overall effectiveness of mitomycin C remains a matter of ongoing debate.^[23–27]^

Synechiae after EDCR most commonly occur between the neo-ostium and anterior half of the middle turbinate. Previous experience with FESS, which is the basis of EDCR and endoscopic nasal surgery, has demonstrated the importance of preventing contact between open wound surfaces to prevent synechiae. Following this pathological basis, various methods are recommended both intraoperatively and postoperatively. The recommended intraoperative procedures are to minimize bleeding and trauma, leave sufficient distance between open wound surfaces, and maintain the integrity of critical structures such as the middle turbinate, ensuring that their stabilization is preserved. If this stabilization is compromised, it must be restored, and nasal packing should be employed. Regular nasal washing with nasal irrigation solutions and cavity dressing are recommended in the postoperative period to prevent synechiae formation in FESS.^[28–30]^

We hypothesize that the middle turbinate-septal suturing technique employed in EDCR prevents direct contact between the raw and denuded surfaces of the area between the neo-ostium and the middle turbinate, thereby reducing the formation of synechiae through a mechanism analogous to that observed in FESS.

The middle turbinate-septal suturing technique offers several additional advantages including improved accessibility for office-based crust removal and postoperative wound care, enhanced visibility for monitoring fistula patency, and facilitation of postoperative washing.

Given the potential benefits of the middle turbinate-septal suturing technique in preventing synechiae, its routine use may be advantageous in EDCR procedures, particularly for patients at an increased risk of synechiae formation, such as those with narrow nasal cavities, excessive intraoperative edema, and intraoperative bleeding. Although our findings offer valuable insights into the potential benefits of the suturing technique, it is important to acknowledge the limitations of this retrospective study, including the lack of a standardized scale for assessing the functional impact of synechiae.

## 5. Conclusion

Suturing the middle turbinate to the nasal septum at the end of surgery reduces the occurrence of postoperative synechiae and contributes positively to surgical success. The middle turbinate-septal suturing technique may be beneficial, particularly in patients with narrow nasal cavities or excessive intraoperative edema and bleeding who are more likely to develop synechiae and granulation tissue formation.

## 6. Overview

This study evaluates the impact of the middle turbinate-septal suturing technique on postoperative synechiae formation and surgical success in EDCR. A total of 98 patients with NLD obstruction who underwent EDCR, and silicone tube intubation were analyzed. Patients were categorized into a suture group and a nonsuture group. The results demonstrated that synechiae and granulation tissue formation were significantly lower in the suture group (*P* = .046 and 0.04). Additionally, both anatomical and functional success rates were significantly higher in the suture group than in the nonsuture group (*P* = .019 and 0.025). These findings suggest that middle turbinate-septal suturing reduces postoperative complications and enhances surgical success.

## Author contributions

**Conceptualization:** Kamil Gokce Tulaci, Tugba Tulaci.

**Data curation:** Kamil Gokce Tulaci, Tugba Tulaci.

**Formal analysis:** Kamil Gokce Tulaci, Tugba Tulaci.

**Investigation:** Kamil Gokce Tulaci, Tugba Tulaci.

**Methodology:** Kamil Gokce Tulaci, Tugba Tulaci.

**Project administration:** Kamil Gokce Tulaci, Tugba Tulaci.

**Resources:** Kamil Gokce Tulaci, Tugba Tulaci.

**Software:** Kamil Gokce Tulaci, Tugba Tulaci.

**Supervision:** Kamil Gokce Tulaci, Tugba Tulaci.

**Validation:** Kamil Gokce Tulaci, Tugba Tulaci.

**Visualization:** Kamil Gokce Tulaci, Tugba Tulaci.

**Writing – original draft:** Kamil Gokce Tulaci, Tugba Tulaci.

**Writing – review & editing:** Kamil Gokce Tulaci, Tugba Tulaci.

**Funding acquisition:** Tugba Tulaci.
